# Supplementary material for: Structure and chemical bonding in high-pressure potassium silver alloys
Source: Commun Chem. 2024 Jul 24;7:162. doi: 10.1038/s42004-024-01245-9 (PMC11269638; doi:10.1038/s42004-024-01245-9)
Supplement: Supplementary file 2 — Supplementary Information [file 42004_2024_1245_MOESM2_ESM.pdf]

Structure and Chemical Bonding in High-Pressure K-Ag Alloys

Nnanna Ukoji[1], Danny Rodriguez[1], Huiyao Kuang[1], Serge Desgreniers[2] and John Tse[1*]

[1] Department of Physics and Engineering Physics

University of Saskatchewan

Saskatoon, Saskatchewan, S7N 5E2, Canada

[2] Laboratoire de physique des solides denses, Department of Physics

University of Ottawa

Ottawa, Ontario, K1N 6N5, Canada

Supplementary Figures

In the following Figures S1- S10, we provide useful information pertaining to the MEM analysis of K_3Ag at five different pressure points. For each pressure, the 2D electron density along the (100) and (110) planes are shown.

The interaction between atoms is indicated by the contour lines encompassing two nearest neighbor atoms. Inspection of the figures show interactions between K-K and K-Ag atoms. In particular, the K-Ag interactions are more discernible in the (100) plane, while the K-K interactions can be seen in the (110) plane.

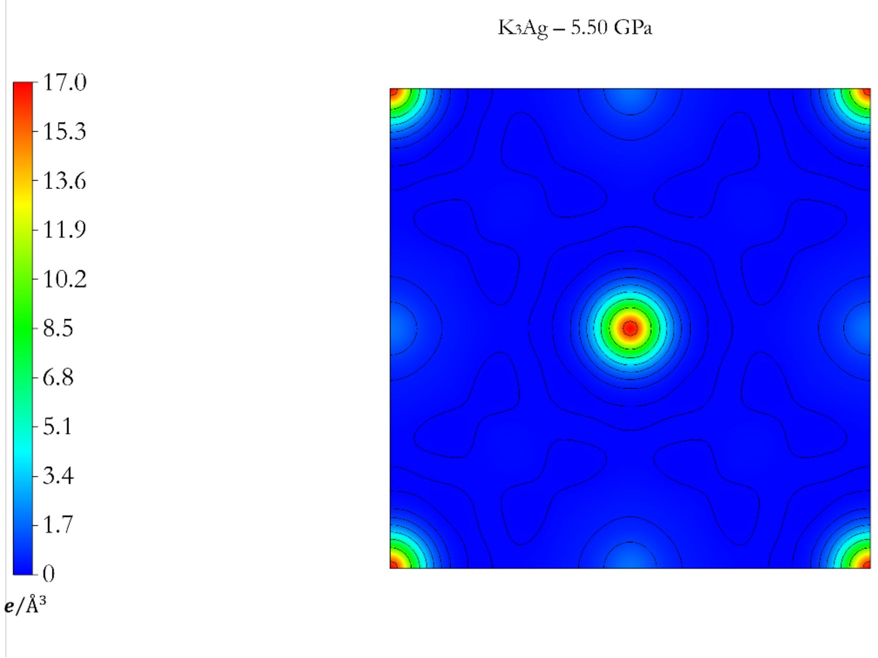

Figure S1. Electron density distribution of K_3Ag in the (100) plane at 5.50 GPa.

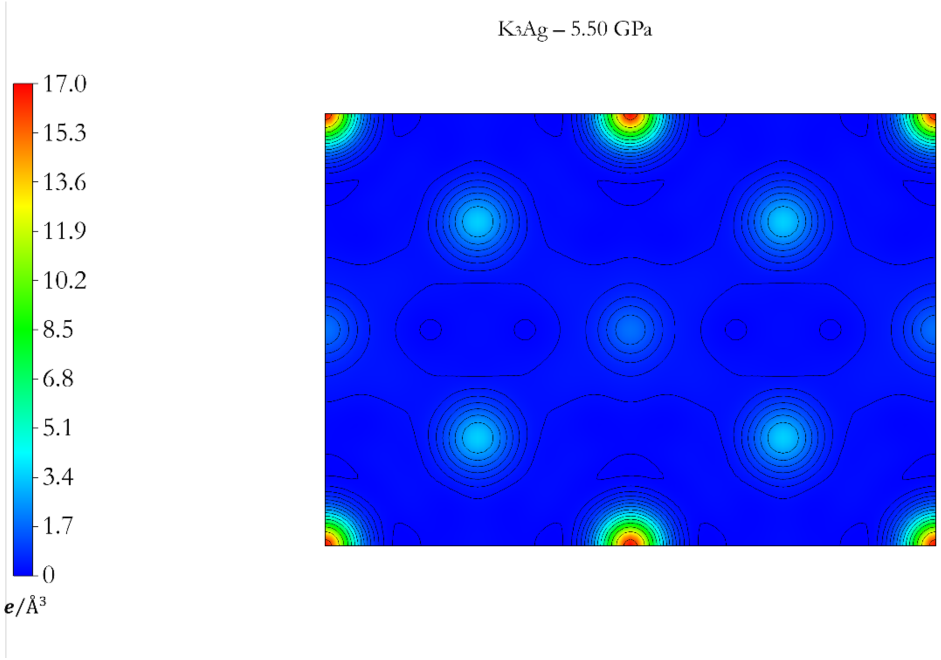

Figure S2. Electron density distribution of K_3Ag in the (110) plane at 5.50 GPa.

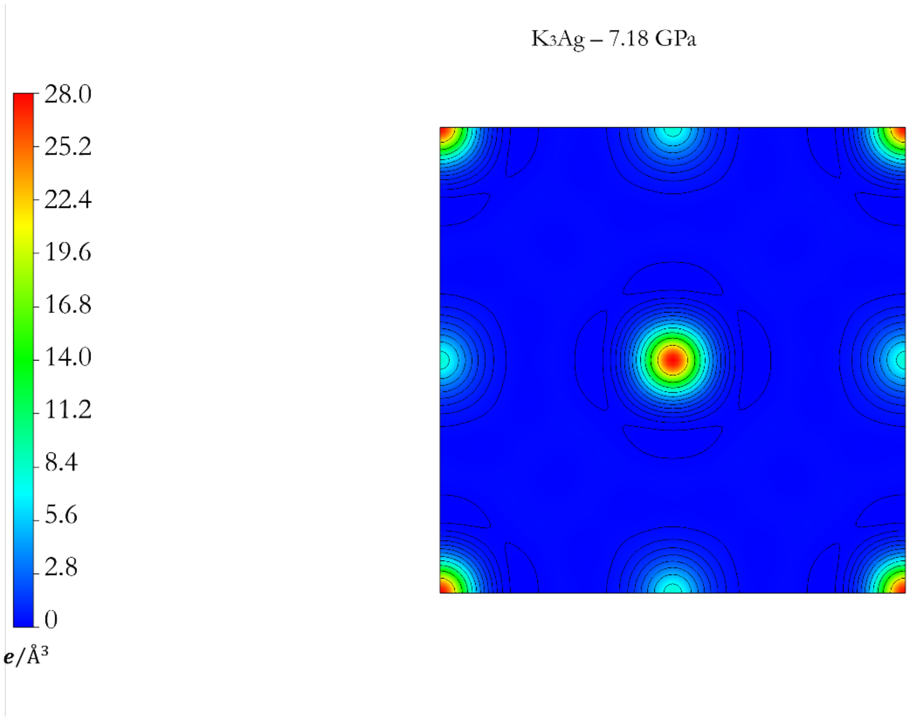

Figure S3. Electron density distribution of K_3Ag in the (100) plane at 7.18 GPa.

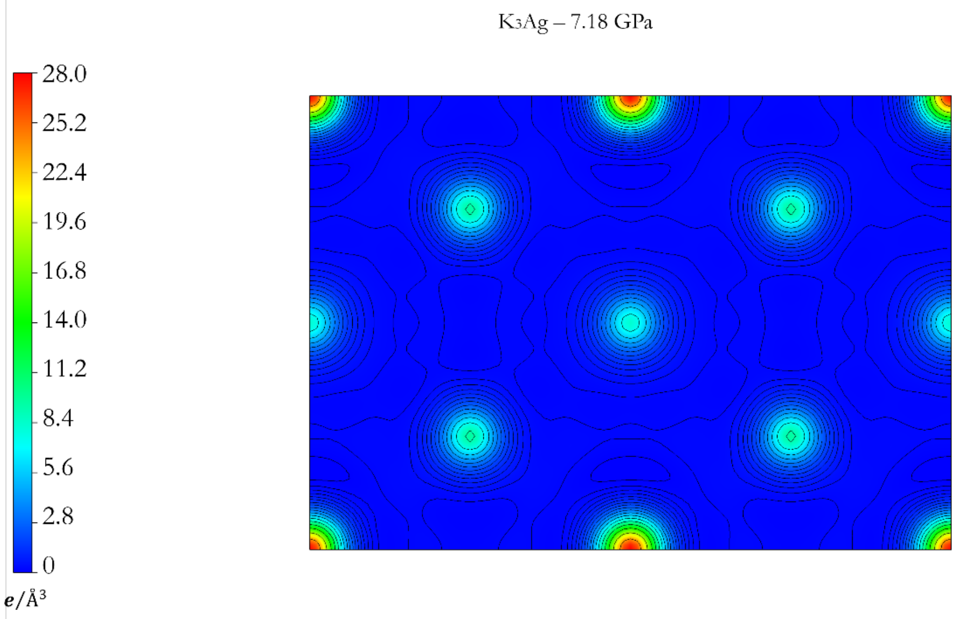

Figure S4. Electron density distribution of K_3Ag in the (110) plane at 7.18 GPa.

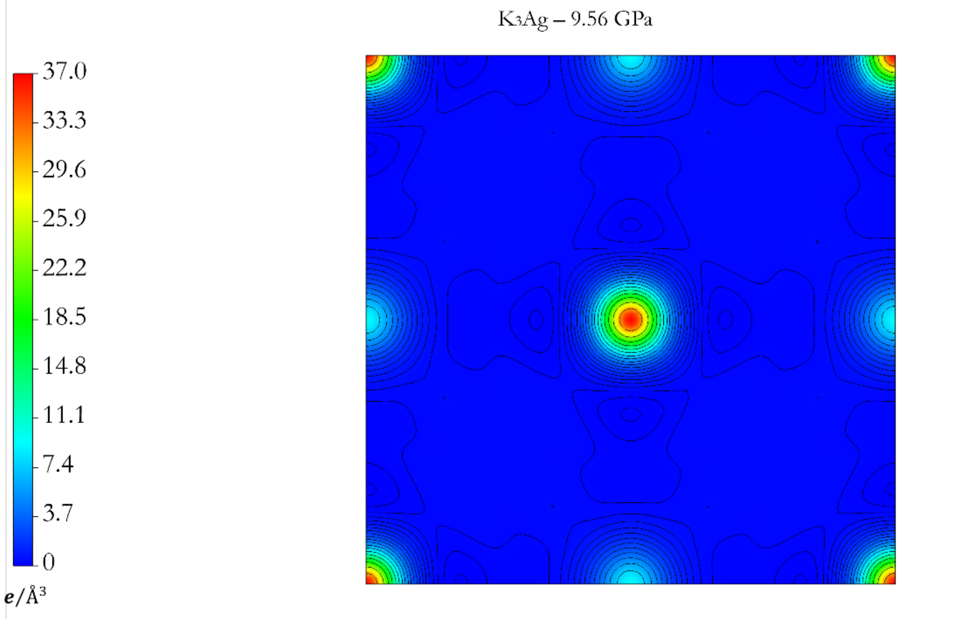

Figure S5. Electron density distribution of K_3Ag in the (100) plane at 9.56 GPa.

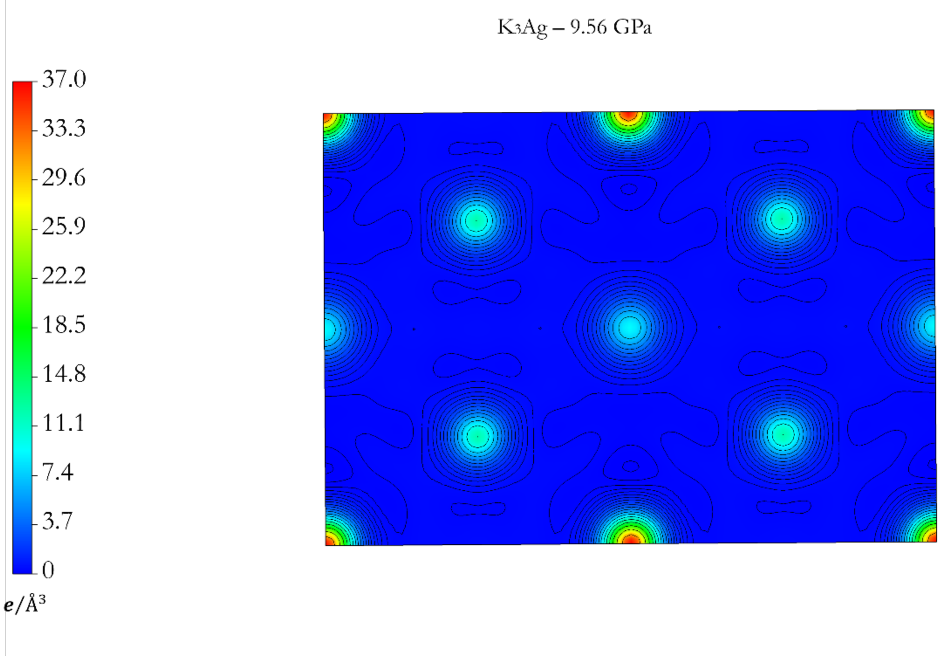

Figure S6. Electron density distribution of K_3Ag in the (110) plane at 9.56 GPa.

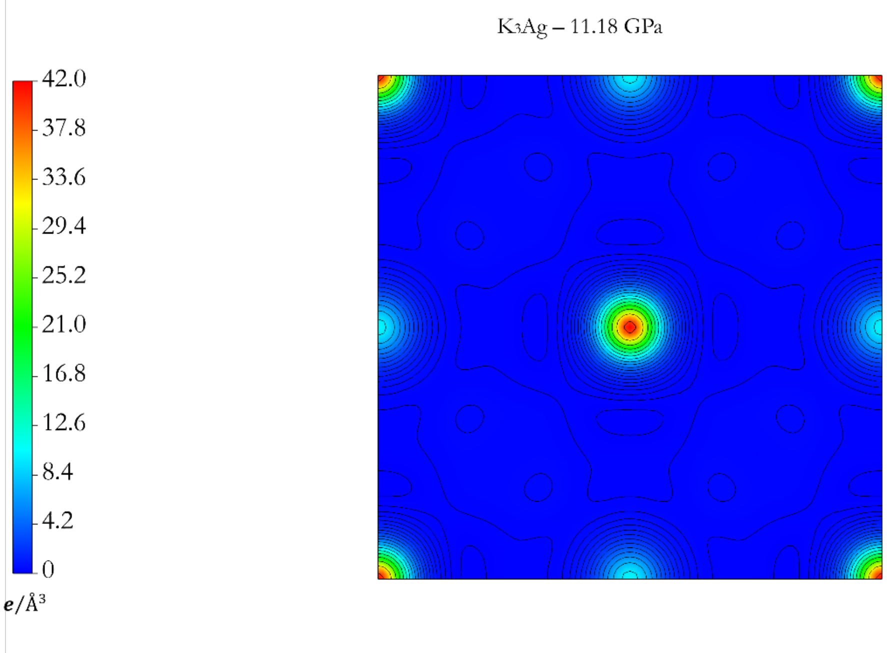

Figure S7. Electron density distribution of K_3Ag in the (100) plane at 11.18 GPa.

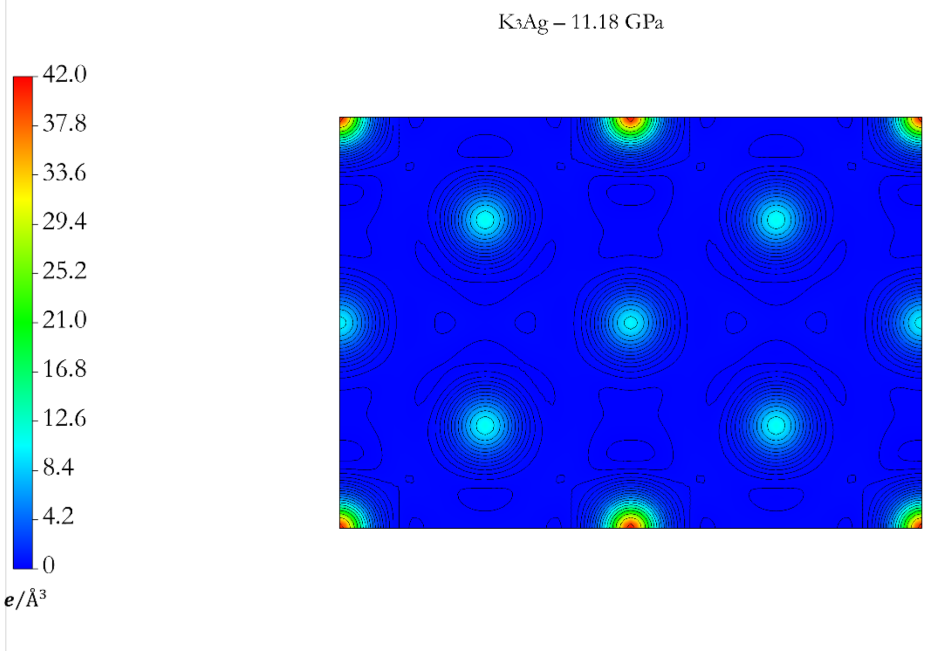

Figure S8. Electron density distribution of K_3Ag in the (110) plane at 11.18 GPa.

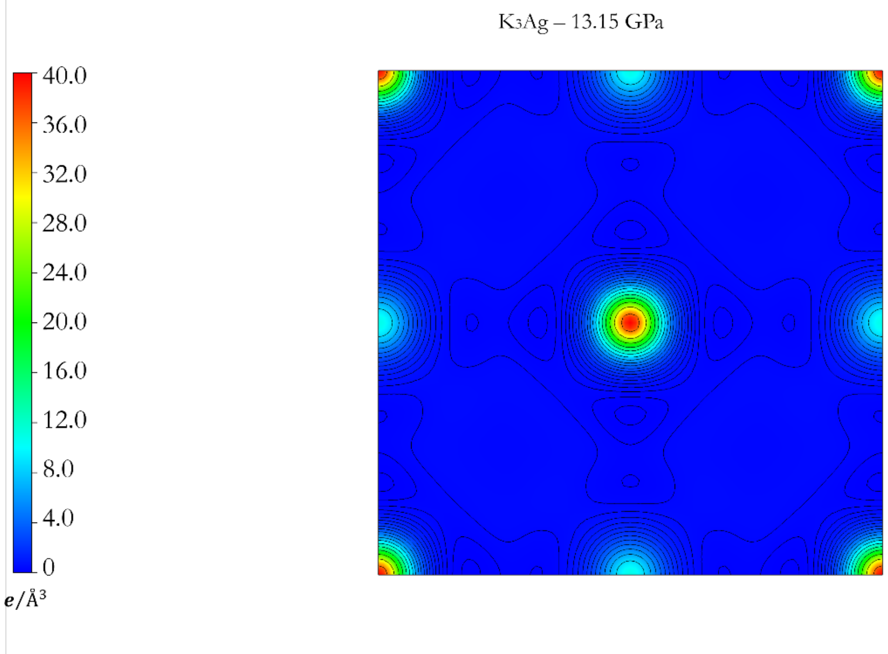

Figure S9. Electron density distribution of K_3Ag in the (100) plane at 13.15 GPa.

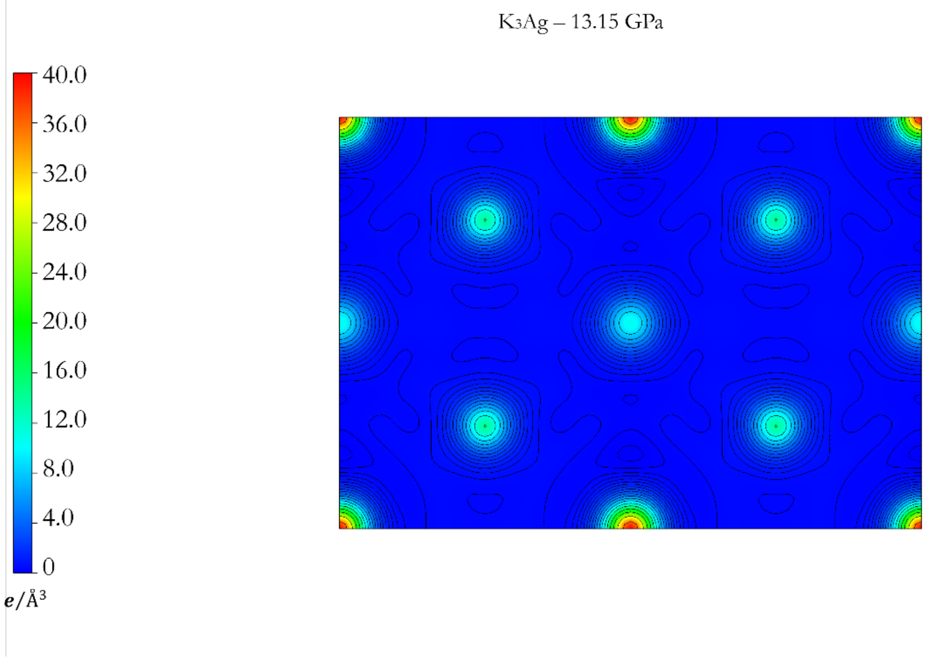

Figure S10. Electron density distribution of K_3Ag in the (110) plane at 13.15 GPa.
